# Supplementary material for: Chromothripsis during telomere crisis is independent of NHEJ, and consistent with a replicative origin
Source: Genome Res. 2019 May;29(5):737–49. doi: 10.1101/gr.240705.118 (PMC6499312; doi:10.1101/gr.240705.118)
Supplement: Supplemental Material [file supp_gr.240705.118_Supplemental_file_1.zip › contigs/annotated_contigs/DB46/contig.2.DB46_length_701_mean_cov_9.7089871612.docx]

**DB46_length_701_mean_cov_9.7089871612**

```
ATTTAAATATAGGTAATTAAAATTTAAATGCAATTTGTTTTTTTTTTTAGAAGGAGTCTCACTCTGTCGCCCAGGCTGGAGTTCAGTGG
 >chr15:58934362-58934712 - E=9e-193 p=0e+00
CGCAATCTCGGCTCACTGCAGCCTCTGCCTTCCAGTTTCAAGCGATTCTCCCACCTCAGCCTCCCGAGTAGCTGGGATTACAGGCGTCC

ACTGTCATGCCCGGCTAATTTTTGTATTTTTGTAGATACAGGGTTTCACCATGTTGGCCAGGCTGGTCTTGAACTCCTGACCTCAGGTG

ATCACCCGCCTCGGCCTCCCAAAGTGCTGGGATTACAGGCGTGAGCCACTGTGACGGGCCTTACATGCAATTTTTATTT|AT|GGTTTC
                                                                                >chr15:
TGATTTTCCTATTATTATTTTTCTGATTTCTCTTAATTGATTTTAGCTGTGAAAGCACTGTCATTTGCTGTTAAATTCTAAAGTCCCTG
58926484-58926790 - E=2e-164
TTAATGAATAGATGAAGTACAAAATGATAAAAATATTTAAGTGGAAATATGGTTTTTCTTTTAAAATTTGATAGTGTCCTAAAGACATA

CTGGAACAATCATAATTCAGTTTTTAATTTTTTTTTGAAGATTTCAAACCTGCGGAAAGGTTGCAAGAATAGTATGGTGAAATTCTTCT

TCTTTTTTTTTTTTTAGACAGATTGTCACT|TTGTTGCCTGGGTTGGGTTTCTGTGGTTCAATCTTAACTCATTGTATCTT
```
